# Recent progress in mucosal immunology and vaccine development

**DOI:** 10.1038/emm.2014.2

**Published:** 2014-03-14

**Authors:** Mi-Na Kweon

**Affiliations:** 1 Mucosal Immunology Section, Laboratory Science Division, International Vaccine Institute, Seoul, Korea

Mucosal surfaces are covered with specialized epithelial cells that serve as physical barriers to the environment and prevent exogenous challenges by pathogens and soluble antigens.^1^ Functionally independent of the systemic immune compartments, the mucosal immune system has developed its own highly organized lymphoid tissues. Mucosal tissues maintain homeostasis by mounting specialized anti-inflammatory immune defenses, such as the production of secretory IgA (SIgA) antibodies and the induction of tolerance against innocuous soluble substances and commensal bacteria. When antigens are administered with appropriate adjuvants or attenuated live vaccines via mucosal routes (oral, nasal, sublingual, ocular, genital or rectal), the mucosal immune system can trigger both humoral and cell-mediated immune protection not only in mucosal sites but also systemically.^2^ Furthermore, owing to mucosal homing properties, local mucosal immunization leads to antigen-specific T- and B-cell responses at both local and distal mucosal sites. Nevertheless, most pathogens can still effectively invade by crossing the host's mucosal membranes. Hence, effective vaccines that exert protective effects at mucosal surfaces are much needed.

The mammalian host has evolved organized secondary lymphoid tissues in the gastrointestinal and upper respiratory tracts that facilitate antigen uptake, processing and presentation for the induction of mucosal immune responses. This is best exemplified by Peyer's patches, which are gut inductive sites that contain a dome, underlying follicles (B-cell zones with germinal centers) and interfollicular regions enriched with T cells. The surface of the dome region is covered by a specialized follicle-associated epithelium that contains microfold (M) cells, which are adept at the uptake and transport of luminal antigens. In addition to serving as transporters for luminal antigens, the M cells also provide an entryway for various pathogens.^3^ After successful uptake, antigens are immediately processed and presented by the underlying dendritic cells (DCs). Therefore, knowledge of the molecular and cellular characteristics of M cells and mucosal DCs is fundamental for the design of mucosal vaccines, including considerations of delivery, adjuvant and formulation. Significant recent advances have been made in the identification of M cell-specific surface markers and the characterization of the M-cell ligand.^4^ As a result, it may now be possible to significantly improve the specificity and efficacy of M-cell-targeting vaccines to elicit both long-lived and protective mucosal immune responses. To date, the targeting of mucosal vaccines to M cells has been successfully performed using M-cell-specific lectins (for example, UEA-1), ligands (for example, complement C5a receptor), antibodies (for example, NKM 16-2-4 and glycoprotein 2) and other proteins (for example, Claudin 4 and reovirus hemagglutinin protein σ1).

DCs are professional antigen-presenting cells that are found at various body sites, where they survey and process microorganisms.^5^ At mucosal interfaces, they are found in the lamina propria, subepithelium, T-cell zones of lymphoid tissue associated with the mucosa and draining lymph nodes.^6^ Mucosal DCs can control the suppressive regulation of commensal bacteria and innocuous antigens and protect the host against pathogens by generating various helper T cells (for example, Th1/Th2/Th17 cells), CD8^+^ T cells and SIgA antibodies. Among the mucosal DC subsets, CD103^+^ DCs play an indispensable role in T-cell differentiation, whereas CX3CR1^+^ DCs can sample and process both circulatory and gut luminal antigens.^7^ Gut CD103^+^CD11b^+^ DCs, TNF-α/iNOS-producing DCs (TipDCs) and TLR5^+^ DCs express retinaldehyde dehydrogenase and thus can be used for IgA class switching. It seems likely that the specific targeting of antigens in a vaccine to specified mucosal DC subsets could result in effective stimulation of T- and B-cell responses.

Because cutaneous and mucosal tissues are constantly exposed to exogenous antigens, each tissue has adopted various types of innate immune cells to facilitate immune surveillance. Mast cells in mucosal compartments (mucosal-type mast cells) are key players in the control of inflammatory, allergic and infectious diseases. In fact, treatment with activators that are specific for mast cells results in protective immunity against pathogens in mucosal compartments.^8^ Mucosal-type mast cells mainly produce histamine, proteases (for example, tryptase and chymase) and TNF-α, all of which may play crucial roles in protection, similar to the effect of an adjuvant. In addition, mucosal-type mast cells are involved in induction of SIgA by IL-6 and in physical interactions via CD40-CD40L. Conversely, downregulation of mast cell activation ameliorates symptoms related to gut allergies, pathogenic infection and inflammation.^9, 10^

Development of therapeutic vaccines that target autoimmune disorders is an important goal. A recent study found that oral vaccination with an attenuated *Salmonella* vaccine strain expressing colonization factor antigen I (CFA/I) fimbriae elicited fimbriae-specific Treg cells without compromising the vaccine's capacity to protect against travelers' diarrhea or salmonellosis.^11^ In this model, enterotoxigenic *Escherichia coli* acquire a plasmid that encodes the fimbriae (referred to as CFA), which mediates *E. coli* colonization in the gastrointestinal tract. This new concept suggests that a vaccine regimen could be designed to stimulate bystander immunity to control autoimmunity, as has been demonstrated in experimental models of autoimmune diseases such as multiple sclerosis and rheumatoid arthritis. The advantage of the oral *Salmonella*-CFA/I model is that autoimmunity could potentially be cured by vaccinating patients with an innocuous antigen, and that protective immunity against enteric bacterial infections could also be induced, thereby ‘killing two birds with one stone.'

In this special feature of *Experimental & Molecular Medicine*, four review articles provide an overview of our current understanding of mucosal immunity and mucosal vaccines.
